# MicroRNA155 Expression in Different Phenotypes and Genotypes of Behçet’s Disease in a Sample of Egyptian Patients

**DOI:** 10.31138/mjr.31.3.337

**Published:** 2020-09-30

**Authors:** Sally S. Hassouna, Manal Y. Tayel, Ashraf I. Alzawawy, Dalal M. Elkaffash, Ahmed M. Abdel Hadi, Eman H. Elsayed, Rowayda M. Amin, Eman Tayae, Alya Elkayal, Asmaa Nasr

**Affiliations:** 1 Internal Medicine Department, Rheumatology and Immunology Unit, Faculty of Medicine, Alexandria University, Alexandria, Egypt; 2 Clinical and Chemical Pathology Department, Faculty of Medicine, Alexandria University, Alexandria, Egypt; 3 Opthalmology Department, Faculty of Medicine, Alexandria University, Alexandria, Egypt

**Keywords:** Behçet’s, microRNA155, phenotypes

## Abstract

**Aim:**

To display microRNA155 (miRNA155) expression in different entities of Behçet’s disease (BD), and to find out if expression is affected in more than one of disease status than another, either phenotypically, according to HLA B51 expression, presence of family history, or patients’ age.

**Methods:**

Thirty BD patients (13 of which were HLAB51 positive) and 15 healthy subjects’ samples were obtained. White blood cell miRNA155 expression in both types of samples was estimated.

**Results:**

Results showed that there is a degree of relation between decrease of miRNA155 expression and different disease aspects, and also, that miRNA155 has an inverse relation with the patients’ ages.

**Conclusion:**

MiRNA155 might be used as a measure of disease of different phenotypes, and that any manifestation of the disease can happen when the expression level decreases.

## BACKGROUND

Behçet’s Disease (BD) is a multi-systemic disorder diagnosed by having oral ulcers more than 3 times/year, and at least two of the following: recurrent genital ulcers, cutaneous rashes, ophthalmic, rheumatologic or neurologic presentation.^1^ The disease is present mainly in the Far East, Middle East and in the silk route countries.^2^ Pathogenesis of the disease is still unknown but is said to be an inflammatory auto immune illness where many genetic and epigenetic changes are shown to be involved and many cytokines are assumed to regulate the pathways of the disease.^2,3^

MicroRNAs (miRNAs) are small, endogenous double-strand RNAs (dsRNAs) that play vital roles through focusing on mRNAs for interpretation or cleavage suppression.^4^

## OBJECTIVE

To display miRNA155 expression in different BD phenotypes, to know if expression is affected more in one disease status than another, and if HLA B51 positivity, presence of family history in patients differ from each other in miRNA155 expression and relation between miRNA155 expression and patients’ ages.

## METHODS

Thirty BD patients and 15 healthy subjects’ blood samples were obtained. White blood cell (WBCs) miRNA155 expression in both sample types was estimated through RNA extraction, reverse transcription, amplification, and then detection by small RNA assays.

**Table 1.** Clinical characteristics of the patients involved.

**Patients’ characteristics**	**No.**	**%**
Oral ulcers	27	90.0
Genital ulcers	25	83.3
Skin lesions:	19	63.3
Ocular involvement:	17	56.7
Rheumatologic complaints:	18	60.0
Vascular events:	7	23.3
Neurological manifestations:	21	70.0
Gastrointestinal symptoms:	3	10.0

## RESULTS AND DISCUSSION

**Figure 1.**
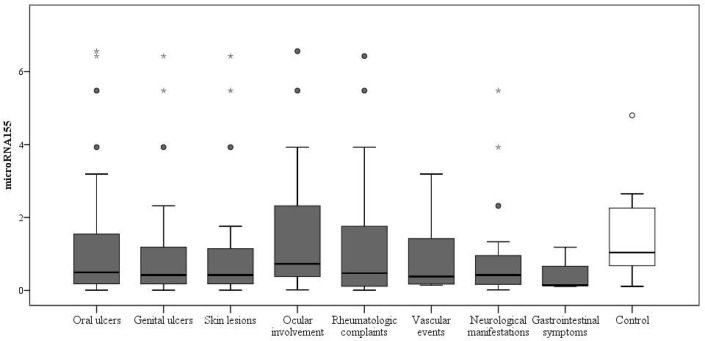
Relation between miRNA155 with different disease clinical presentations.

**Figure 2.**
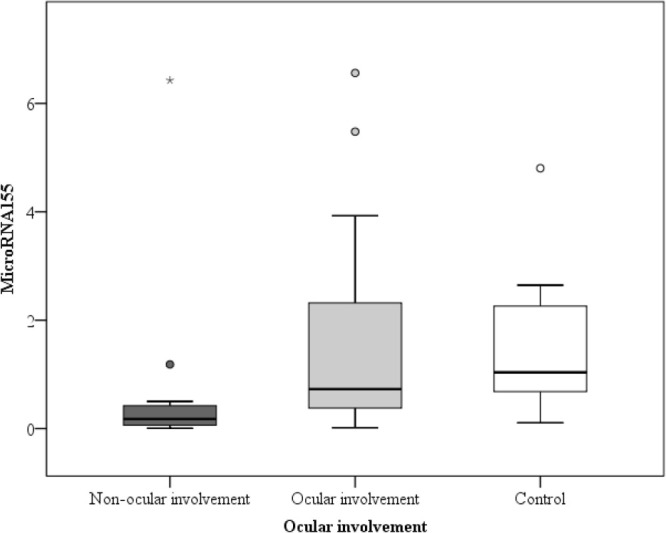
MiRNA155 expression in ocular and nonocular BD.

MiRNA155 expression levels were decreased non-significantly not only in the whole patients group compared to the control group, but also decreased non-significantly in all disease clinical presentations except for those not having eye problems who significantly had a decreased expression from the control group p= 0.013, and from patients with ocular involvement (p=0.041) (**Figures 1** and **2**).

HLA B51 positivity in involved patients was 43.3%, and there was no significant difference between miRNA155 expression in either HLA positive or HLA B51 negative patients and the control group (p = 0.201).

**Figure 3.**
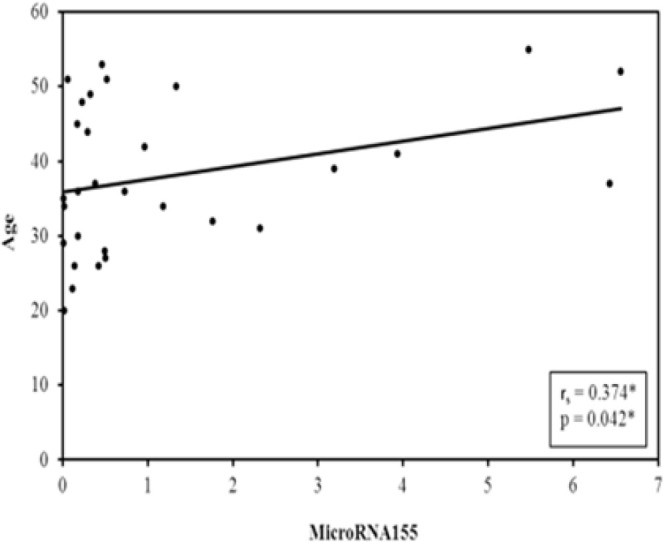
Correlation between miRNA155 expression and the patients’ age.

Also, there was no significant difference between miRNA155 expression in patients having positive family history of BD (7 patients) and those who did not (p=0.081). MiRNA155 expression appeared to be directly proportional to age (rs=0.374, P=0.042) (**Figure 3**): this may be explained by decreased disease activity with increasing age. These findings prove what was mentioned in several studies, that miRNA155 decreases with increase of BD activity, such as Hassouna et al.^5^

The results shown are coherent with researches telling that increase in miRNA155 is related to decreased expression in some cytokine, eg, interleukin (IL)1, IL 6 and IL 17, as is shown in Zhou et al.^6^ These ILs play important roles in BD pathogenesis, as noted in many studies, eg, Yamakawa et al.^7^ and Emmi et al.,^8^ and this increased miRNA155 expression leads to increased expression of inhibitory IL 10,^6^ and results in a healthy immune system and may lead to balance of macrophages (M1/M2) through IL13 receptor alpha targeting (Martinez-Nunez et al.).^9^ A study showed that high level of MiRNA155 was found in Tregs in a FOXP3-dependent way, and Treg homeostasis is disabled in miRNA155 insufficiency. When miRNA155 is missing in Treg, miRNA155 target of cytokine signalling 1 expression suppression increase with diminished responses to IL2, which is an imperative Treg homeostasis controller this was found by Lu et al.^10^ Moreover, it was discovered by Ceppi et al. that miRNA155 down-regulates lipopolysaccharide induced inflammatory pathways through inhibition of monocyte-derived DCs.^11^

A few other researches revealed an inverse result to ours, that miRNA155 expression is expanded in activity of BD, and that MiR-155 focusing on Ets-1 controls Th17 response and that concealment of miR-155 diminishes pathogenic IL-17-expressing T cells, as was seen in the study of Na et al.^12^ Another research found that T cells miR-155 lack in mice did not lead to advancement of extreme autoimmune encephalomyelitis (EAE) and has a diminished IL-17 generation which is a critical cytokine for EAE pathogenesis.^13^

Moreover, Blüml et al.^14^ found that mice lacking in miRNA155 have impedance in Th17 differentiation and in turn are protected from joint inflammation initiated by collagen.

The opposite findings to our research results shown in some other studies may be due to ethnic differences, and different activity levels of the disease in the involved patients from one study to another. Also, several studies (either those having same results to ours, or the contrary) were built on animal research, which cannot be relied on. Thus, there is a controversy about miRNA155 role in the pathogenesis of BD disease.

## CONCLUSION

MiRNA155 might be used as a measure of disease state. Any manifestation of the disease can happen when the expression level decreases.

## References

[1] Sakane T Takeno M Suzuki N Inaba G BD disease. N Engl J Med 1999;34 1:1284–91. 10.1056/NEJM199910213411707 10528040

[2] Shahriyari E Vahedi L Roshanipour N Jafarabadi MA Khamaneh A Laleh MG Exploring the association of IL-10 polymorphisms in Behcet’s disease. J Inflamm 2019;16:26. 10.1186/s12950-019-0230-2 PMC6929502 31889911

[3] Morton LT Situnayake D Wallace GR Genetics of Behçet’s disease. Curr Opin Rheumatol 2016;28:39–44. 26599381 10.1097/BOR.0000000000000234

[4] O’Connell RM Rao DS Chaudhuri AA Baltimore D Physiological and pathological roles for microRNAs in the immune system. Natr Rev Immunol 2010;10:111–22. 10.1038/nri2708 20098459

[5] Hassouna SS Tayel MY Elkaffash DM Abdelhady AM Elsayed EH MicroRNA155 Expression in Relation to BDCAF Scored Behҫet’s Disease in an Egyptian Patients’ Sample. Open Rheumatol 2018;12:115–22. 10.2174/1874312901812010115 PMC6110073 30197703

[6] Zhou Q Xiao X Wang C Zhang X Li F Zhou Y Decreased microRNA-155 expression in ocular BD disease but not in Vogt Koyanagi Harada syndrome. Invest Ophthalmol Vis Sci 2012;53:5665–74. 22815348 10.1167/iovs.12-9832

[7] Yamakawa Y Sugita Y Nagatani T Takahashi S Yamakawa T Tanaka S Interleukin-6 (IL-6) in patients with BD disease. J Dermatol Sci 1996;11:189–95. 8785169 10.1016/0923-1811(95)00439-4

[8] Emmi G Talarico R Lopalco G Cimaz R Cantini F Viapiana O Efficacy and safety profile of anti-interleukin-1 treatment in BD disease: a multicenter retrospective study. Clin Rheumatol 2016;35:1281–6. 26156661 10.1007/s10067-015-3004-0

[9] Martinez-Nunez RT Louafi F Sanchez-Elsner T The interleukin 13 (IL-13) pathway in human macrophages is modulated by microRNA-155 via direct targeting of interleukin 13 receptor alpha1 (IL13Ralpha1). J Biol Chem 2011;286:1786–94. 21097505 10.1074/jbc.M110.169367 PMC3023473

[10] Lu LF Thai TH Calado DP Chaudhry A Kubo M Tanaka K Foxp3-dependent microRNA155 confers competitive fitness to regulatory T cells through targeting SOCS1. Immunity 2009;30:80–91. 19144316 10.1016/j.immuni.2008.11.010 PMC2654249

[11] Ceppi M Pereira PM Dunand-Sauthier I Barras E Reith W Santos MA MicroRNA-155 modulates the interleukin-1 signaling pathway in activated human monocyte-derived dendritic cells. Proc Natl Acad Sci USA 2009;106:2735–40. 19193853 10.1073/pnas.0811073106 PMC2650335

[12] Na SY Park MJ Park S Lee ES MicroRNA-155 regulates the Th17 immune response by targeting Ets-1 in BD disease. Clin Exp Rheumatol 2016;34:S56–63. 27156371

[13] O’Connell RM Kahn D Gibson WS Round JL Scholz RL Chaudhuri AA MicroRNA-155 promotes autoimmune inflammation by enhancing inflammatory T cell development. Immunity 2010;33:607–19. 20888269 10.1016/j.immuni.2010.09.009 PMC2966521

[14] Blüml S Bonelli M Niederreiter B Puchner A Mayr G Hayer S Essential role of microRNA-155 in the pathogenesis of autoimmune arthritis in mice. Arthritis Rheum 2011;63:1281–8. 21321928 10.1002/art.30281

